# Assessing the Impact of Afforestation on Soil Organic C Sequestration by Means of Sequential Density Fractionation

**DOI:** 10.1371/journal.pone.0117897

**Published:** 2015-02-23

**Authors:** Weiwei Cong, Tusheng Ren, Baoguo Li

**Affiliations:** 1 Department of Soil and Water Sciences, China Agricultural University, Beijing, China; 2 Department of Rural Regional Development, Shenyang Agricultural University, Shenyang, Liaoning, China; Fudan University, CHINA

## Abstract

Afforestation is a prevalent practice carried out for soil recovery and carbon sequestration. Improved understanding of the effects of afforestation on soil organic carbon (SOC) content and dynamics is necessary to identify the particular processes of soil organic matter (SOM) formation and/or decomposition that result from afforestation. To elucidate these mechanisms, we have used a sequential density fractionation technique to identify the transfer mechanisms of forest derived C to soil fractions and investigate the impact of afforestation on SOC sequestration. Surface soil samples from continuous maize crop land (C4) and forest land (C3), which had been established 5, 12 and 25 yr, respectively, on the Northeast China Plain were separated into five density fractions. SOC, nitrogen (N) concentration and δ13C data from the three forests and adjacent cropland were compared. Afforestation decreased SOC concentration in the < 2.5 g cm-3 fractions from 5 yr forest sites, but increased SOC content in the < 2.0 g cm-3 fractions from 25 yr forest sites. Afforestation did not affect soil mass distribution, SOC and N proportional weight distributions across the density fractions. The < 1.8 g cm-3 fractions from 12 and 25 yr forests showed higher C/N and lower δ13C as compared to other fractions. Incorporation of forest litter-derived C occurred from low density (< 1.8 g cm-3) fractions to aggregates of higher density (1.8-2.5 g cm-3) through aggregate recombination and C transport in the pore system of the aggregates. Some forest litter-derived C could transfer from the light fractions or directly diffuse and adsorb onto mineral particles. Results from this study indicate that microaggregate protection and association between organic material and minerals provide major contribution to the SOC sequestration in the afforested soil system.

## Introduction

Carbon emission from land use conversion during the last two centuries is approximately 100~150 Pg C [1], and around one half of which is attributed to loss of soil organic matter (SOM) [2]. Conversion of forest to agricultural land can result in the depletion of SOM, while afforestation of agricultural land can, to some extent, promote the long-term C sequestration [3]. For example, China carried out several forest restoration projects, including the Three-North Protective Forest Program and Natural Forest Conservation Program, and sequestered approximately 0.45 Pg C of living biomass from 1970 to 1998 [4, 5]. According to the Kyoto Protocol of 1992, secondary forest area conservation is considered an important method for atmospheric CO_2_ reduction and improvement of C sequestration in the biomass and soil in terrestrial ecosystems. However, the accumulation of SOM can be reversed if forest management is improperly maintained [6]. Previous land management [7], climate characteristics, and the quantity and quality of C input can influence soil C turnover rates following forest establishment [8, 9]. Studies focused on soil organic carbon (SOC) stocks in temperate zone have shown that there was an initial decrease followed by a gradual increase in topsoil SOC stock after afforestation [10–13]. Thus, improved understanding of the effects of afforestation on SOC content and dynamics is necessary to accurately characterize the particular processes of SOM formation and decomposition that result from changes in land use.

However, quantitative investigating the changes in SOC stocks is challenging because the soil matrix is a complex system that consists of fractions with chemical compositions, contributions from plant and microorganisms, and dynamic characteristics vary considerably [14]. A prevalent method of analysis is sonication of the soil sample for disaggregation which separates the soil into different fractions. Acid hydrolysis is also used to generate a liable and stable C fraction which is then combined with stable C isotopes to quantify carbon dynamic characteristics of the separated fractions [15, 16]. The differences in stable C isotope signatures (ca. -27‰ of C_3_ and ca. -11‰ of C_4_) lead to litter and SOC with distinctive isotope signatures [17]. The proportion of newly added SOC and decay rates of old SOC in different fractions can be evaluated by the mixing model [18]. Studies using the above methods showed that afforestation in cropland affected the amount and dynamic rates of old SOC fractions [16, 19], and resulted in significant accumulation of newly added C associated with microaggregates (53–250 μm), silt and clay (< 53 μm) [20]. However, information about interactions between SOM and minerals in the separated fractions cannot be obtained by using the methods previously discussed. The disaggregation method fails to separate the soil into fractions with different mineralogy and SOM composition, and sonication causes SOM redistribution within aggregates [21–23]. Recent work on forest systems suggests that soil fractions separated by way of sequential density fractionation (SDF) presented significant differences in both mineralogy and SOM composition [21, 22]. SOC associated with denser particles showed lower C/N, higher δ^13^C and δ^15^N compared to SOC associated with light particles. Radiocarbon dating indicated that C associated with denser particles typically has a longer turnover time [22]. It is unclear whether SOC in the fractions separated by SDF have distinct dynamic characteristics in response to afforestation.

In Jilin Province, Northeastern China, a deciduous forest was established twenty-five years ago on soil previously used for maize cultivation. Many continuous maize fields were maintained adjacent to the forest sites. We isolated five different density fractions, ranging from < 1.8 to 2.8 g cm^-3^, for the 0–5 cm depth horizon from 5, 12 and 25 years after forest planting and from the crop plots which were adjacent to each forest plot. The objectives of this study were to identify the mechanisms by which forest OC is incorporated into different soil fractions and investigate the impact of afforestation on SOC sequestration in the various fractions.

## Materials and Methods

### Site description

**Fig 1:**
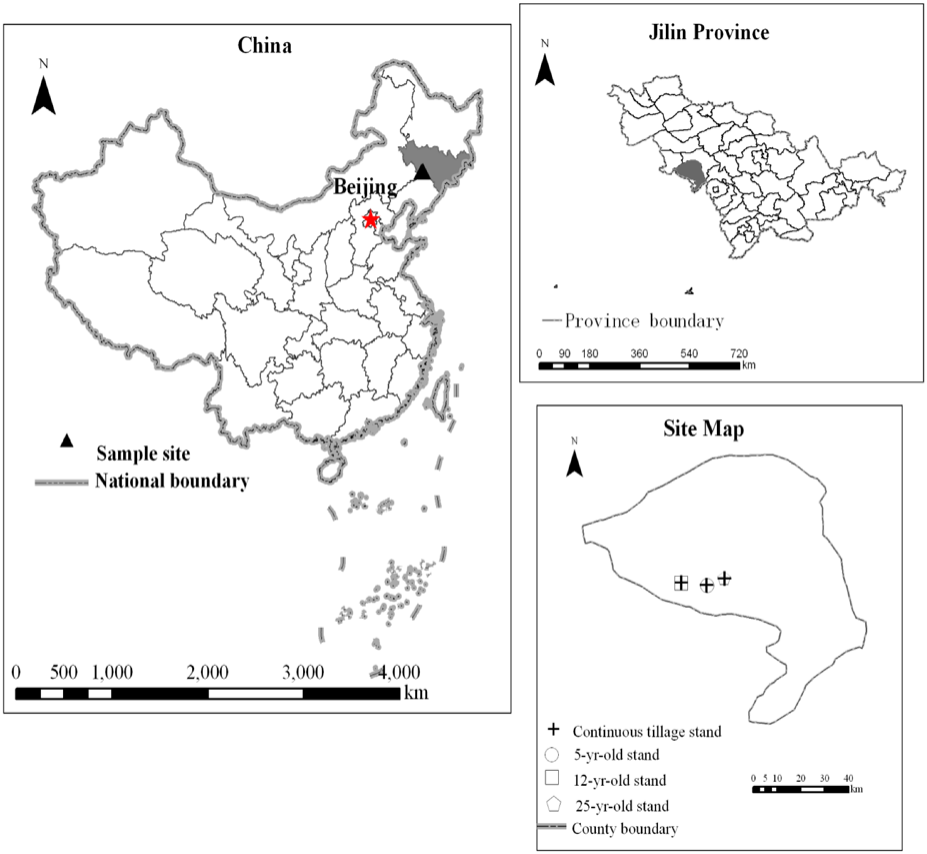
Forest site and adjacent cropland site locations (Jilin province, China).

The study sites (43°19′N, 124°13′E, elevation 200 m) are located at Lishu County of Jilin Province, approximately 1157 km northeast of Beijing (Fig. 1). The authority of study sites is under jurisdiction of the Jilin Province, Lishu County government and all sampling was conducted with permission. The study did not involve endangered or protected species, and the specific location of study is given in Table 1. The long-term annual mean temperature was 5.8°C and annual mean precipitation was 554 mm. Three croplands (labeled “C”) and adjacent forestlands (labeled “F”) were selected (Table 1). The physical and chemical properties of the soil in the paired lands are listed in Table 2. The croplands were under continuous maize (*Zea mays L*.) cultivation for approximately forty years. The forestlands had been converted from croplands for 5, 12 and 25 yr, respectively. Except for the 12 yr forestland and cropland (F12 and C12), where the soils are Fluvents (FAO *Fluvisol*) with a sandy texture, the soils of the other plots were Chernozems (FAO *Calcic Chernozem*) and had a sandy loam texture.

**Table 1:** Sample code, depth, land use, specific location and soil type.

	Code					
	C5	F5	C12	F12	C25	F25
Land use	Cropland	5 yr forest	Cropland	12 yr forest	Cropland	25 yr forest
Soil type	Calciustoll(FAO *Calcic Chernozem*)	Calciustoll (FAO *Calcic Chernozem*)	Fluvent (FAO *Fluvisol*)	Fluvent (FAO *Fluvisol*)	Calciustoll (FAO *Calcic Chernozem*)	Calciustoll (FAO *Calcic Chernozem*)
Location	43°19′22″N, 124°13′50″E	43°19′19″N, 124°13′48″E	43°19′51″N, 124°8′1″E	43°19′42″N, 124°8′2″E	43°20′51″N, 124°17′52″E	43°20′33″N, 124°17′48″E

**Table 2:** Characteristics of bulk soils.

Variable			Soil^a^			
	C5	F5	C12	F12	C25	F25
Sand (%)	70.4 ± 3.2	70.4 ± 4.3	92.4 ± 5.3	94.4 ± 3.1	74.4 ± 3.1	75.2 ± 6.3
Silt (%)	15.2 ± 2.9	14.8 ± 3.8	3.6 ± 0.6	2.4 ± 0.6	14 ± 0.9	13.2 ± 0.7
Clay (%)	14.4 ± 2.6	14.8 ± 3.6	4.0 ± 0.5	3.2 ± 0.4	11.6 ± 3.8	11.6 ± 2.7
pH (H_2_O)	7.7 ± 1.6	8.0 ± 1.7	7.0 ± 0.9	7.2 ± 1.2	7.9 ± 1.3	7.9 ± 0.9
(CaCl_2_)	6.7 ± 1.3	7.0 ± 1.4	6.0 ± 0.9	6.4 ± 1.2	6.8 ± 1.3	6.9 ± 0.9
CEC NH_4_Ac pH_7_ (cmol/kg)	29.2 ± 3.2	27.8 ± 3.3	16.4 ± 2.1	14.3 ± 2.4	21.4 ± 4.1	21.4 ± 3.2
Fe_oxalate_ (g/kg)	1.5 ± 0.8	0.9 ± 0.6	1.6 ± 0.7	1.5 ± 0.3	1.1 ± 0.7	1.0 ± 0.6
Fe_dithionite_ (g/kg)	4.1 ± 1.2	4.4 ± 1.1	3.8 ± 0.9	3.4 ± 0.7	5.0 ± 1.3	5.1 ± 1.5
Al_oxalate_ (g/kg)	1.5 ± 0.9	1.5 ± 0.7	1.0 ± 0.6	0.8 ± 0.7	0.3 ± 0.3	1.1 ± 0.6
Al_dithionite_ (g/kg)	1.3 ± 0.5	1.2 ± 0.3	1.1 ± 0.3	1.2 ± 0.3	1.0 ± 0.4	1.3 ± 0.1
Si_oxalate_ (g/kg)	1.4 ± 0.7	1.4 ± 0.6	0.5 ± 0.2	0.5 ± 0.3	0.6 ± 0.3	0.8 ± 0.3
Si_dithionite_ (g/kg)	3.4 ± 0.7	3.3 ± 0.6	2.4 ± 0.3	2.4 ± 0.3	2.3 ± 0.9	2.3 ± 0.7
CaCO_3_ (g/kg)	0.9 ± 0.1	0.9 ± 0.1	0.5 ± 0.1	0.4 ± 0.1	0.8 ± 0.1	0.8 ± 0.1
TOC (g/kg)	16.7 ± 1.2*	14.1 ± 1.1*	9.7 ± 1.1*	11.6 ± 1.0*	12.9 ± 0.5*	13.9 ± 0.3*
N (g/kg)	1.9 ± 0.1*	1.7 ± 0.1*	1.2 ± 0.2	1.3 ± 0.1	1.4 ± 0.1	1.3 ± 0.2
C/N	8.8 ± 0.1*	8.3 ± 0.2*	8.1 ± 0.9*	8.9 ± 0.3*	9.2 ± 0.5*	10.7 ± 0.6*
δ^13^C (‰)	-22.9 ± 0.7**	-25.6 ± 0.5**	-19.5 ± 0.4**	-23.7 ± 0.5**	-18.2 ± 0.4**	-22.8 ± 0.3**

^a^,: mean of five sites ± standard error.

*: the difference between forest soil and adjacent crop soil was significant at P < 0.05.

**: the difference between forest soil and adjacent crop soil was significant at P < 0.01.

The three croplands (C5, C12 and C25) were established in 1972, 1969 and 1972, respectively. After harvesting, crop residue was removed from the fields, leaving roots in the soil. For C5, C12 and C25, the fertilization rate was 900, 700, and 500 kg N ha^-1^ (as urea), 500, 500 and 400 kg P ha^-1^ (as triple-superphosphate), and 100, 150 and 80 kg K ha^-1^ (as K_2_SO_4_), respectively.

The three deciduous forest (*Populus tomentosa Carr*.) lands (F5, F12 and F25) adjacent to the corresponding croplands were converted from cropland in 2003, 1997 and 1984, respectively. For F5, F12 and F25, the forest area was approximately 3, 6 and 10 ha, respectively. The mean tree trunk diameter, height and tree density of F5, F12 and F25 were 12.1, 20.1 and 32.1 cm, 14.1, 16.1 and 27.6 m, and 805, 810 and 710 stems ha^-1^, respectively.

### Soil sampling

Soil samples were collected in October 2009 following maize harvesting. We employed a paired plot design (afforested land vs. crop land) for comparison. With the assumption that the cropland represented the baseline from which any change in SOM would be attributed to afforestation. We also ignored seasonal variation in SOM content and composition, although it is difficult to select randomly located replicate sites of the same afforestation years with similar soil type, texture and management practices. The main objective of this research is the quantification of carbon changes in a field-scale but not in county-, state- and national-scale. Therefore, our research was centered on analysis of soil C variability within field sites. The microplots were considered as replicated treatments. This method is efficient in detecting changes in soil C in grasslands [24]. To minimize any effect of spatial heterogeneity and potential lack of independence associated with sampling, each block was equally divided into five microplots and sampling sites were placed randomly at least 5 m from one another. The smallest plot area was 60 m×60 m. We did not choose the edge sites to void of the effect of margin. Samples were collected at a 0–5 cm depth where the greatest SOC change usually occurs near the soil surface following afforestation [25]. In each microplot, five replicated samples were collected at a 0–5 cm depth and passed through an 8-mm sieve at field moisture, and the samples were then air-dried for sequential density fractioning. Another three replicated samples were collected at a 0–5 cm depth, mixed thoroughly, air-dried, passed through a 2-mm sieve, and ball-milled to a fine powder for physical and chemical analysis. The soil physical and chemical properties of the paired lands are listed in Table 2. Litter of the forest on the O horizon (on the surface) was the major contributor to forest SOM at the 0–5 cm depth, which was mixed by forest and grass litter. Therefore, litter samples from the O horizon of each forest block were collected and mixed for subsequent analysis. For the cropland sites, root litter samples were collected from the 0–10 cm layer, as the major vegetation input to the SOM originated from maize roots. Two composite litter samples were collected from each site.

### Physical and chemical characterization

The particle size distribution of soil samples was measured via pipette method [26]. Soil pH (in double-deionized water or 0.01 M CaCl_2_) was measured with a glass electrode in the supernatant of a 2.5:1 solution to soil suspension. Cation exchange capacity (CEC) was measured using the BaCl_2_ method [27]. Soil CaCO_3_ was measured using quantitative gravimetric analysis [28].

Oxalate-extractable Fe (Fe_oxalate_), Al (Al_oxalate_) and Si (Si_oxalate_) were determined as following the methodology established by Schwertmann [29]. 2 g of air-dried soil was added to 100 ml acid ammonium oxalate solution (0.2 M ammonium oxalate and 0.2 M oxalic acid) and shaken for 4 h in the dark. The suspension was centrifuged and the concentration was determined by ICP-AES.

Dithionite-extractable Fe (Fe_dithionite_), Al (Al_dithionite_) and Si (Si_dithionite_) were determined as described by Blakemore et al. [27]. About 1-g of the air-dried soil sample was shaken with 1-g sodium dithionite and 50 ml sodium citrate (22%) for 16 h. After adding 10 ml of 0.05 M MgSO_4_ flocculent, the sample was centrifuged, and the supernatant was decanted. The supernatant was constituted with 100 ml with double-deionized water, and the concentration was determined with ICP-AES.

### Sequential density fractionation

Soil density fractions were isolated according to the method described by Sollins et al [22] and 5 fractions were selected as follows: < 1.8, 1.8–2.0, 2.0–2.5, 2.5–2.8 and > 2.8 g cm^-3^. Lithium heteropolytungstates (LSTs, Central Chemical Consulting Pty Ltd) with low C (< 0.001%) and N concentration (< 0.001%) were used for repeated separation. 5 air-dried samples (15 g each) were immediately set aside to determine soil water content. Three subsamples (30 g each) were placed in 225 ml polycarbonate centrifuge tubes and shaken initially for 30 min on a shaking table. According to Sollins et al [21], this step disperses weakly bound aggregates and preserves fine scale aggregates. Because the soil used was air-dried and not the moist bulk sample, we shook it for 30 min to avoid dispersion aggregates. The soil suspensions were centrifuged at 2400 rpm (970 g) in a swinging bucket rotor for 20 min. The light fraction (supernatant) was aspirated and rinsed with deionized H_2_O on a glass fiber filter (Whatman GF/F, 0.7 μm particle retention) until the density of washing liquid (< 1.01 g cm^-3^) was obtained. The heavy fraction (residue at the bottom of the centrifuge tubes) was added to next higher density LST and adjusted to the target density (± 0.01 g cm^-3^), shaken (30 min) and the procedure repeated. The cleaned, sequentially isolated fractions were oven-dried (55°C for 48 h). Dried fractions were weighed and ball-milled to a fine powder. Fractions from the three subsamples were mixed into one sample for subsequent laboratory analysis. All values were calculated using the oven-dried values. Mass recovery was > 95% for all samples.

### Pretreatment of soil sample

For OC, N, and stable carbon isotope analysis, soil sample was treated with HCl vapor to remove CaCO_3_ [30]. The powdered sample was then oven dried (60°C for 48 h), and a 200–300 mg subsample was added to a 20 ml glass weighing bottle and moistened using 150 μl deionized water. The bottle was moved into a pyrex glass vacuum desiccator (7.5 l) fitted with a porcelain plate, together with a beaker containing 100 ml of 12 M HCl. The desiccator was vacuum sealed (at 80 kPa) using a vacuum pump and the samples were exposed to HCl vapor for 48 h. The beaker with HCl was then removed, the desiccator resealed, and the samples subjected to repeated vacuum evacuation for 1–1.5 h to remove HCl vapor. The samples were then dried (60°C for 16 h) and allowed to cool to room temperature in a desiccator prior to manual grinding (mortar).

### Physical appearance

Density fraction power (< 0.2 mm) was visually observed with a stereo microscope (Nikon SMZ800). Petri dishes were gently hand shaken and pincers were used to check for the presence of different density phases. Samples were checked for recognizable organic debris and mineral particles.

### Mineralogy

Mineralogy of the fractions was assessed by X-ray diffraction (XRD). Soil samples were hand-ground with mortar and pestle to a mean particle size < 200 μm, then back loaded into a 2.5 cm diameter circular cavity holder and run on a PANanalytical X’Pert Pro instrument using Co Kα radiation at 40 kV and 40 mA. Diffraction patterns were recorded by step scanning from 3–70° 2θ, with the sample spinning at 2 revolutions per second. Phase identification and peak area determinations were executed using the X’pert High Score plus software.

### Total organic carbon (TOC) and total N

TOC and total nitrogen N content was determined by way of the dry combustion method using an elemental analyzer (Flash EA1112, CE Instruments, Wigan, UK). Analytical precision was ± 0.1 mg g^-1^ for SOC content and ± 0.05 mg g^-1^ for N content. Recovery rate was > 90% of SOC for all soils and was > 85% of total soil N for all soils.

### Carbon isotope ratio analysis

Stable carbon isotope composition was determined with a Delta^Plus^ XP mass spectrometer coupled to an elemental analyzer in continuous flow mode. The combustion temperature of the elemental analyzer was 1020°C. The isotope ratio was expressed as *δ* ^13^C (‰):

δ13C=[(RsampleRstandard)−1]/1000 (1)

where *R* is the molar ratio of the heavy to light isotope of the sample or standard (ViennaPeedee Belemnite, V PDB). The precision for isotope measurements was ± 0.1‰. The *δ* ^13^C values of the SOM and fractions were used to calculate the proportion of forest C (C_3_) from the mass balance equation [20], with the assumption that there was no shift in the C_3_/C_4_ input ratio in the cropped soils over the years. The calculations were completed for each forest soil fraction following Eq. (2):

$$F=\left(\delta_{T}-\delta_{crop\;soil}\right)/\left(\delta_{forest\;litter}-\delta_{crop\;soil}\right)$$ (2)

where *F* is the fraction of SOC originating from the current forest litter, *δ*_T_ the *δ* ^13^C value of the current total SOC in forest, *δ*_crop soil_ the *δ* ^13^C value of the total SOC in the crop soil at the specified fraction, *δ*_forest litter_ the *δ* ^13^C value of the forest litter (C_3_).

New C, indicating SOC concentration originating from the forest litter (C_3_), and old C, the SOC concentration from the crop litter, were calculated using Eq. (3) and Eq. (4), respectively,

New C=F×TOC (3)

Old C=(1−F)×TOC (4)

The variable *δ*_T_, *δ*_crop soil_, and *δ*_forest litter_ are independently measured and the source variability should considered in *F* calculation. Standard errors (SE) of *F* can be calculated using partial derivatives [18] as:

$$\sigma_{F}^{2}=\frac{1}{{\left(\delta_{forest\;litter}-\delta_{crop\;soil}\right)}^{2}}\left[\sigma_{\delta_{T}}^{2}+F^{2}\sigma_{\delta_{forest\;litter}}^{2}+{\left(1-F\right)}^{2}\sigma_{\delta_{crop\;soil}}^{2}\right]$$ (5)

where *σ* ^2^_δ crop soil_, *σ* ^2^_δ forest litter_ and *σ* ^2^_δ T_ represent variances of the mean *δ*_crop soil_, *δ*_forest litter_ and *δ*_T_, respectively. The *σ*_F_ is the SE of the proportion (*F*) estimated [18]. All calculations were performed by the tools provided by Phillips and Gregg [18] at http://www.epa.gov/wed/pages/models.htm.

The decomposition rates constants (*k*) for the old C of different fractions were calculated calculated following Del Galdo et al [20]:

$$\begin{array}{c} k=-\text{ln}(\frac{\text{Old C}}{\text{TOC}})/t\\ =-\text{ln}\left(1-F\right)/t\\ =-\text{ln}\left(F'\right)/t \end{array}$$ (6)

where *k* is the net relative decomposition rates constants of old C, *t* is the age of forest (year) and *F’* is the proportion of old crop C. The standard error of *k* is calculated based on the SE of the proportion (*F’*) estimated [31]:

$$\text{Δ}k=f^{\prime }\left(F^{\prime }\right)\Delta F^{\prime }+\frac{f^{\prime \prime }\left(F^{\prime }+\theta \Delta F^{\prime }\right)}{2!}{\left(\Delta F^{\prime }\right)}^{2},\theta \in \left(0,1\right)$$ (7)

where Δ*F’* is standard error of *F’*.

### Statistical analysis

Data were presented as the means of the five replicates with standard errors. Analysis of variance (one-way ANOVA) was carried out for TOC, total N and *δ* ^13^C measurements per soil fraction. Significant differences in TOC, total N and *δ* ^13^C between forest soil and the adjacent crop soil were tested using the least square difference (LSD) test (p < 0.05) after confirming normal distributions and homogeneity of variance. All analyses were carried out using the SPSS software.

## Results

### Physical appearance

**Fig 2:**
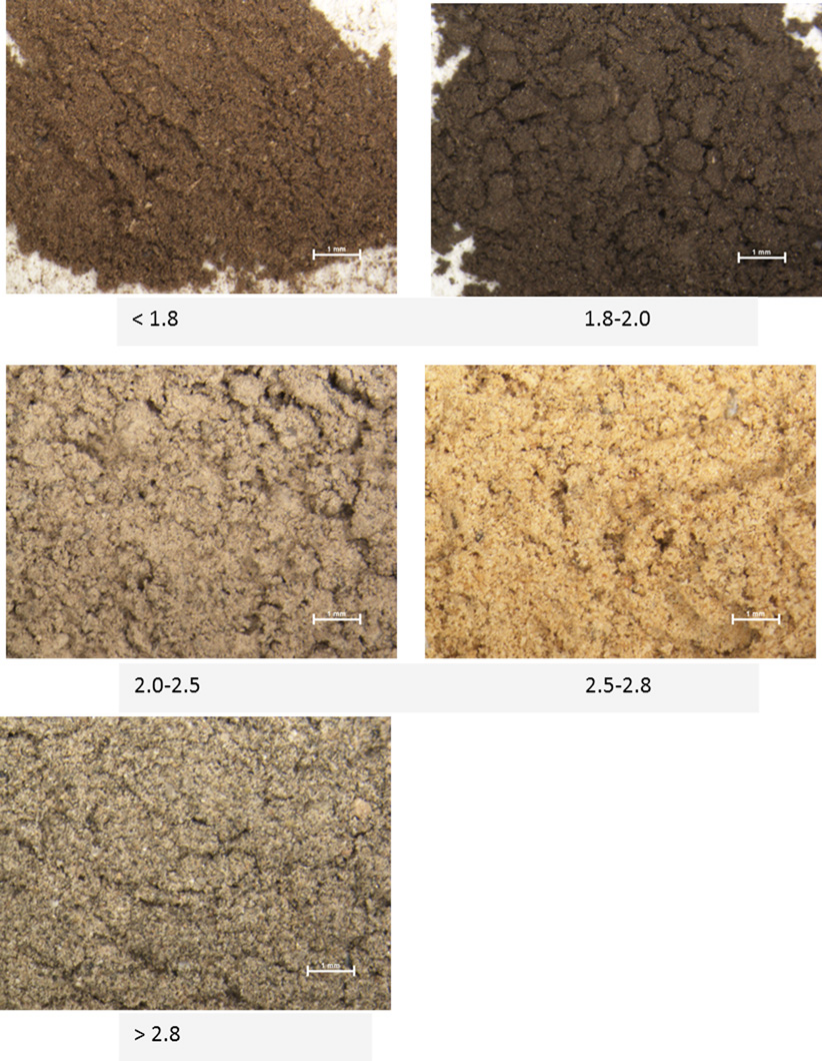
Light microscope images of 5 density fraction powders.

Microscope observations confirmed that the physical appearance of the same density fractions varied much among all crop and forest sites (Fig. 2). The fraction < 1.8 g cm^-3^ consisted of plant debris and trapped minerals. The 1.8–2.0 g cm^-3^ fractions consisted of adsorbed OM or microaggregates and some minerals. The 2.0–2.5 g cm^-3^ fractions consisted of adsorbed OM, microaggregates and large amount of entrapped minerals. The > 2.5 g cm^-3^ fractions were mainly grains. The 2.5–2.8 g cm^-3^ fractions exhibited a yellow to brown color, while the fractions > 2.8 g cm^-3^ were a dark green color.

### Mineralogy

**Fig 3:**
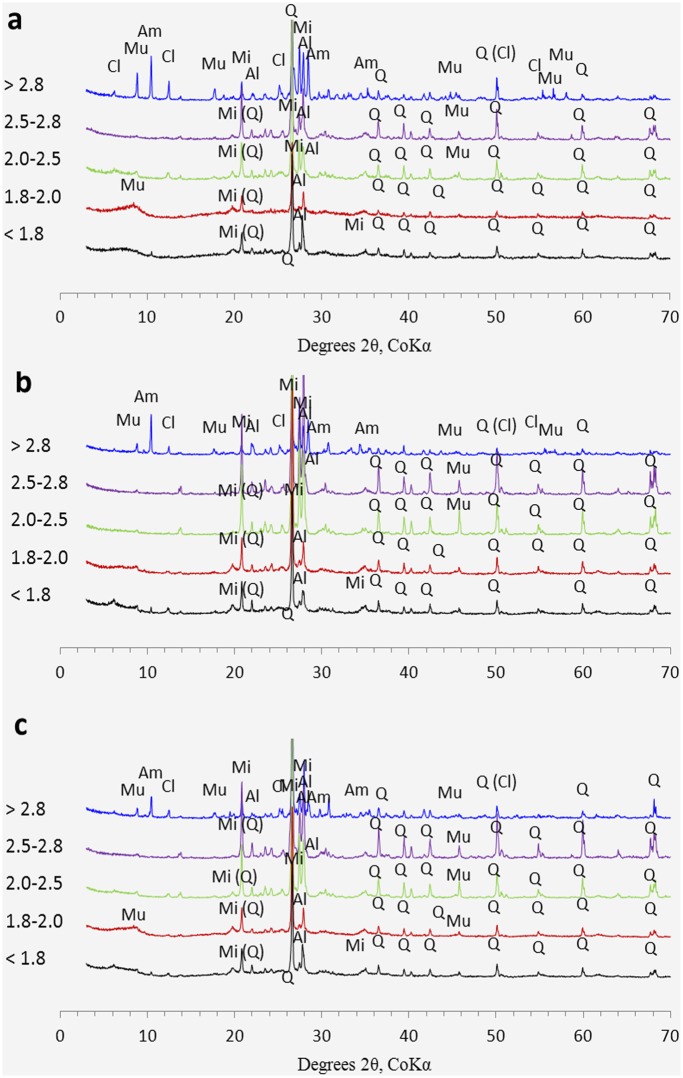
X-ray diffraction traces for the density fractions from each soil. A. 5 year old forest; b. 12 year old forest; c. 25 year old forest. Al: albite; Am: amphibole; Cl: clinochlore; Mi: microcline; Mu: muscovite; Q: quartz. For a specific density fraction, soil samples from the Crop lands showed the same X-ray diffraction traces with that of adjacent forest soil. Therefore, only the results for forest soils were presented.

All soils showed similar patterns for XRD scans and suggested that their mineralogies were similar despite large differences in texture (Fig. 3): Fractions < 2.0 g cm^-3^ showed the presence of quartz, albite and microcline. The broad peak between 6° and 10° 2*θ* of the 1.8–2.0 g cm^-3^ fractions is associated to somewhat poorly crystalline clinochlore and muscovite. This broad feature was not present in the > 2.0 g cm^-3^ fractions. The 2.0–2.5 g cm^-3^ fractions presented dominant components of quartz (2.65 g cm^-3^), albite (2.6–2.7 g cm^-3^), microcline (2.53–2.56 g cm^-3^) and muscovite (2.8–2.9 g cm^-3^). The 2.5–2.8 g cm^-3^ fractions showed the maximum signal intensity of quartz and albite. In the > 2.8 g cm^-3^ fractions, quartz was less prominent than the lighter fractions and is replaced by the albite, amphibole (3.2–3.4 g cm^-3^), clinochlore (2.3–3.4 g cm^-3^) and muscovite (2.8–2.9 g cm^-3^).

### SOC, N content and distribution

**Fig 4:**
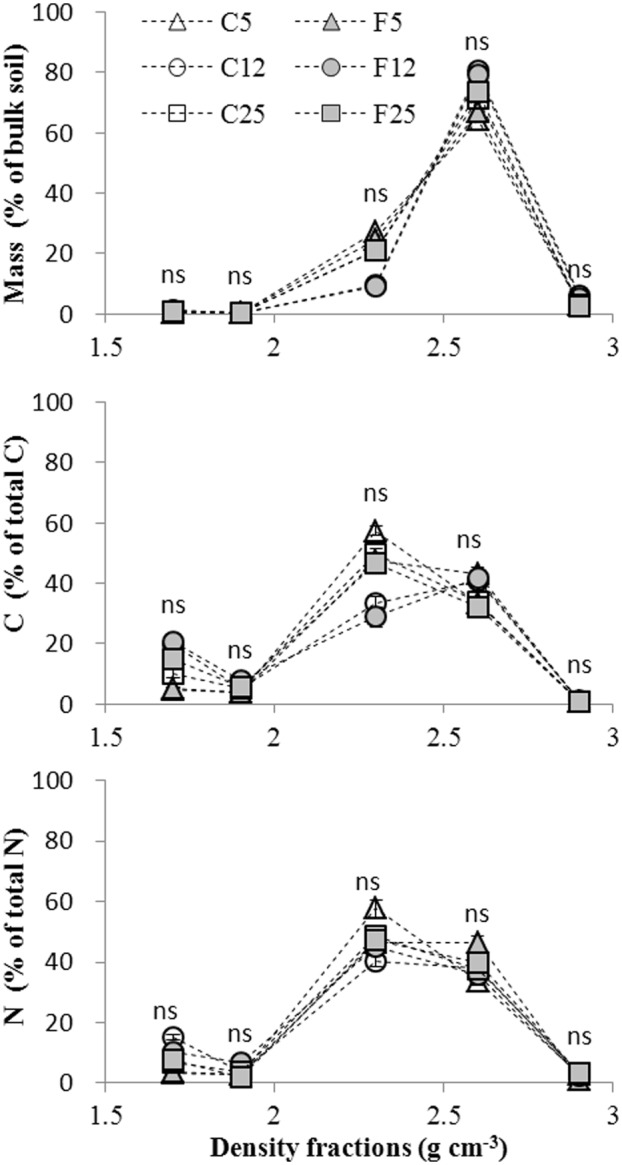
Distribution of mass, SOC and N across the density fractions from the crop and forest land (F5, 5 yr forest; C5, F5 adjacent crop soil; F12, 12 yr forest; C12, F12 adjacent crop soil; F25, 25 yr forest; C25, F25 adjacent crop soil. Error bars are standard error of the means; ns: P > 0.05. The density axis in this graph reflects the midpoint of each density range except for the two extremes which are plotted as the lowest and highest minus or plus 0.1 g cm^-3^.

**Fig 5:**
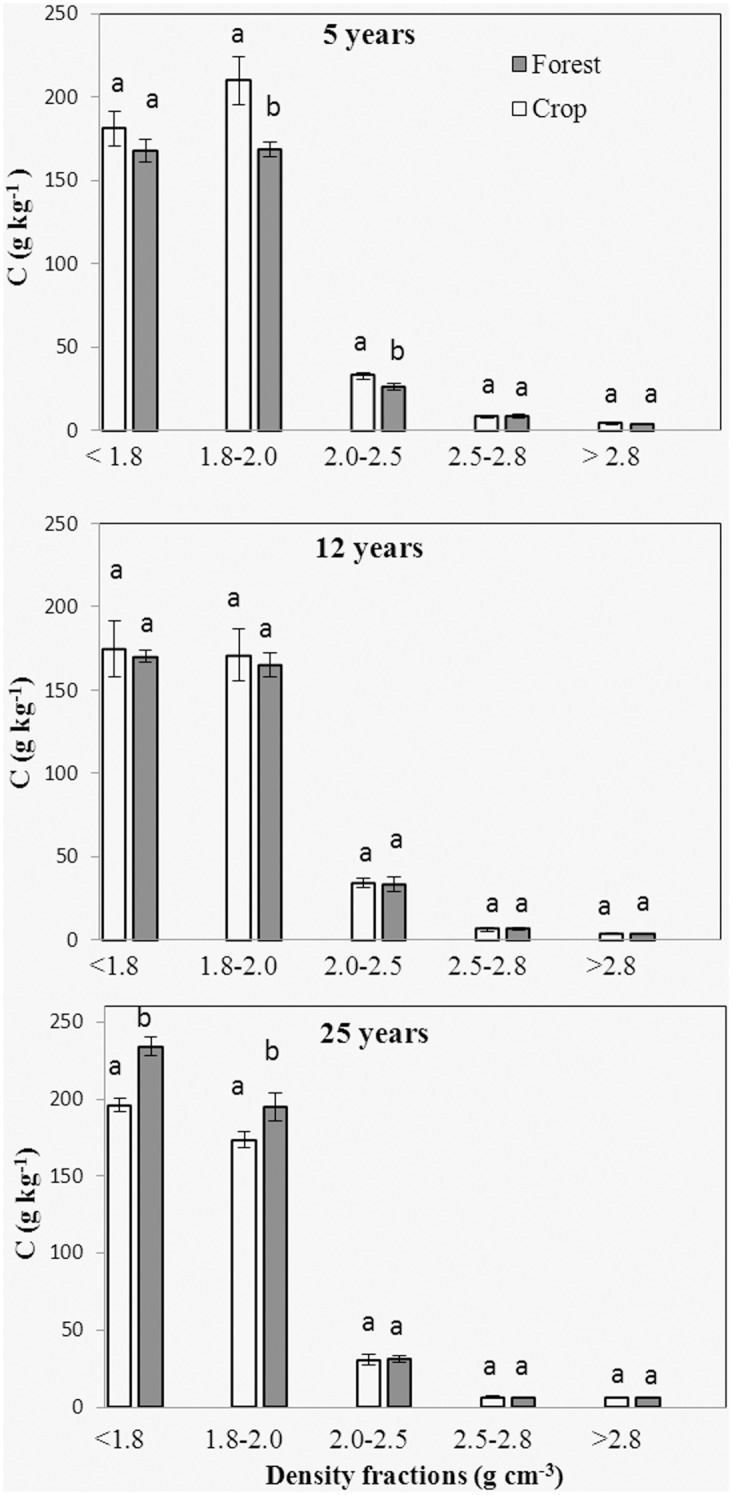
SOC concentration for the 5 density fractions from 5, 12 and 25 yr forest and the adjacent crop soil. Error bars are standard error of the means; Within a group, different letters indicate a significant difference between land uses (P < 0.05).

**Fig 6:**
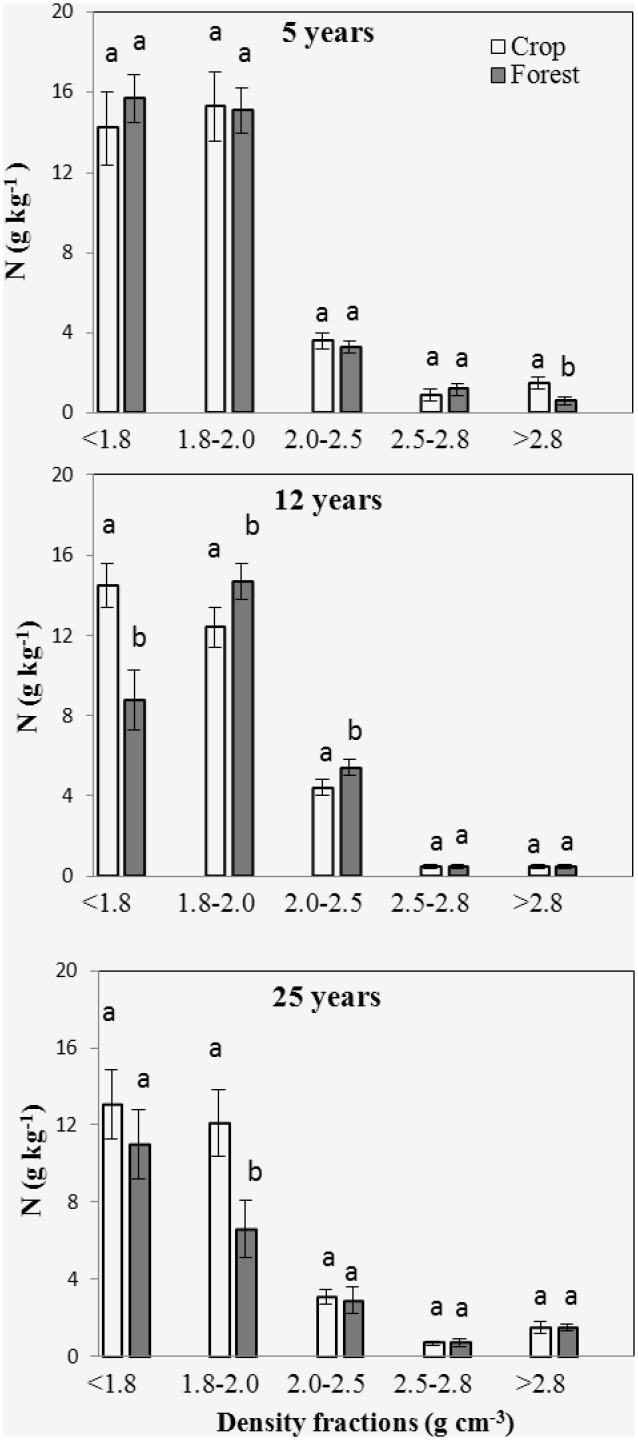
Total N concentration for the 5 density fractions from 5, 12 and 25 yr forest and its adjacent crop soil. Error bars are standard error of the means. Within a group, different letters indicate a significant difference between land uses (P < 0.05).

**Fig 7:**
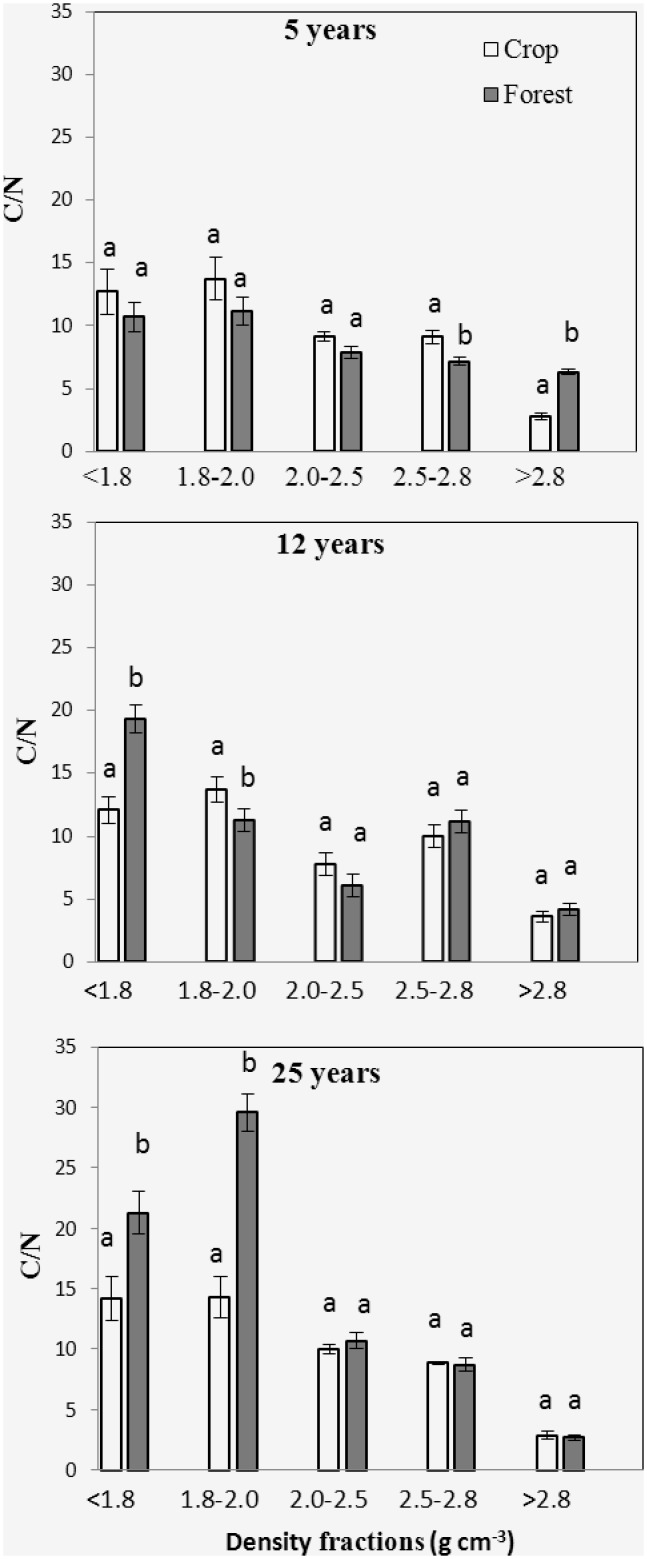
C/N for the 5 density fractions from 5, 12 and 25 yr forest and its adjacent crop soil. Error bars are standard error of the means. Within a group, different letters indicate a significant difference between land uses (P < 0.05).

No significant differences were observed between forest and adjacent soil in the distributions of soil mass, SOC and N (Fig. 4). Dry weight strongly peaked in the 2.5–2.8 g cm^-3^ fraction, which accounted for 60% of total soil mass. Mass proportions for the remaining fractions ranged from 1–20% of total soil mass. The SOC and N distributions showed different patterns from that of density fraction mass distribution. SOC and N peaked in the 2.0–2.5 g cm^-3^ fraction, which made up 40% of total SOC and N. At both forest and crop sites, SOC, N concentrations and C/N dropped from the light fraction to the heavy fractions, except for the C12 and F12 sites, and there was a slight increase of C/N in the 2.5–2.8 g cm^-3^ fraction (Figs. 5–7).

Significant differences in SOC contents were observed between the bulk forest soil and adjacent crop soil (Table 2). The SOC content of the F5 site was lower than that of the C5 site, while SOC contents of the F12 and F25 sites were higher than that of the C12 and C25 sites. Comparing to the C5 sites, soil N content was reduced at the F5 site. No significant differences were observed in N contents between F12 and C12, and between F25 and C25. The disproportional decrease in SOC with respect to total N resulted in a decline in C/N at the F5 compared with C5. In contrast, the disproportional increase in SOC with respect to total N at the F25 site produced significantly higher C/N ratio than that of the C25 site. No significant C/N difference was found between F12 and C12.

Isolated fractions from forest and adjacent crop soil samples had different SOC and N concentrations (Figs. 5–6). SOC content decreased in the 1.8–2.5 g cm^-3^ fractions of the forest site F5, but increased in the < 2.0 g cm^-3^ fraction from F25 compared with the adjacent crop soil, respectively. No significant difference in SOC content was observed between F12 and adjacent crop soil for all the density fractions. N concentration was lower in the fraction > 2.8 g cm^-3^ of the forest site F5 compared with the adjacent crop soil. N concentration was lower in the < 1.8 g cm^-3^ fraction, but higher in the 1.8–2.5 g cm^-3^ fraction from the forest site F12 compared with the adjacent crop soil. At forest site F25, N concentration decreased in the fractions 1.8–2.0 g cm^-3^ compared with the adjacent crop soil. A downward trend in C/N ratios was observed from the 1.8–2.0 g cm^-3^ to the 2.0–2.5 g cm^-3^ fraction. The densest fractions hold the lowest C/N in comparison to other fractions. Comparing with the adjacent crop land, C/N increased in the > 2.8 g cm^-3^ fraction from F5, in the < 1.8 g cm^-3^ fraction from F12 and in the < 1.8 and 1.8–2.0 g cm^-3^ fractions from F25, respectively (Fig. 7). C/N decreased in the 2.5–2.8 g cm^-3^ fraction from F5 and the 1.8–2.0 g cm^-3^ fraction from F12 compared to the adjacent crop soil, respectively.

### δ ^13^C of density fractions

**Fig 8:**
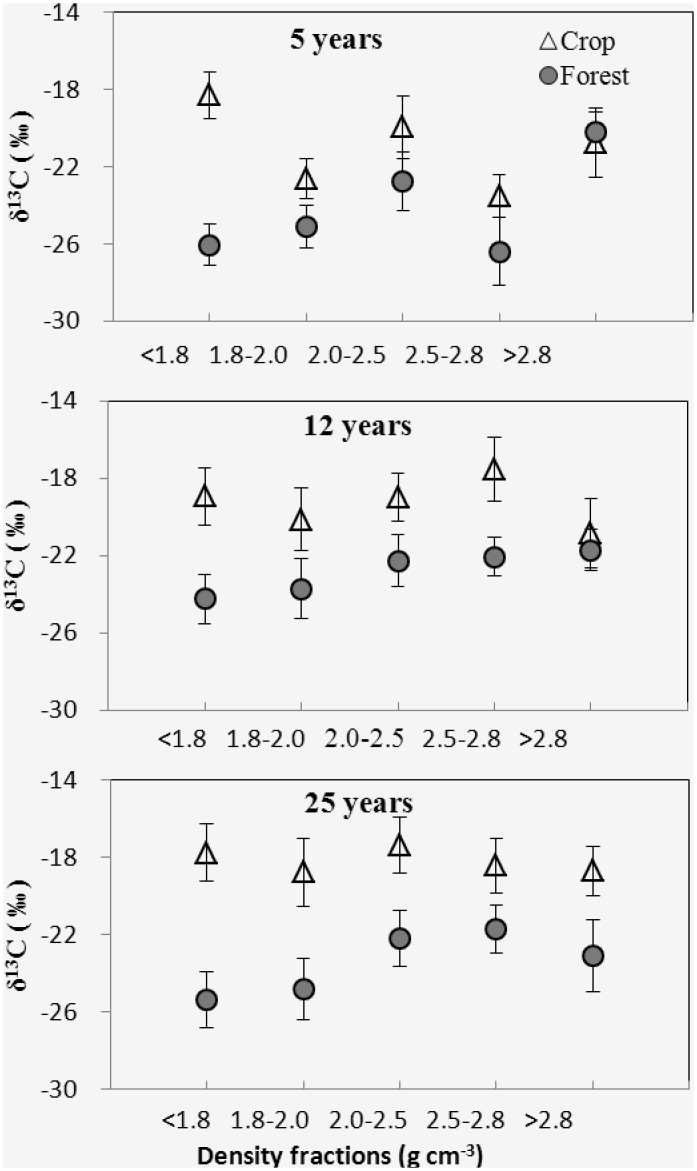
δ^13^C for the 5 density fractions from 5, 12 and 25 yr forest and adjacent crop sites. Error bars are standard error of the means. The δ^13^C data for arable crop litter and forest litter were-12.8‰ and-29.0‰, respectively.

The *δ* ^13^C values for crop litter and forest litter were-12.79‰ and-28.97‰, respectively. The *δ* ^13^C values of soil OM ranged between-26.37‰ and-17.36‰. In the 5 yr forest sites, δ^13^C values decreased in the < 1.8 and 1.8–2.0 g cm^-3^ fractions compared to the corresponding fractions from crop soil (Fig. 8). In the 12 yr forest sites, except for the > 2.8 g cm^-3^ fraction, the δ^13^C values decreased in other fractions. The δ^13^C values of the F25 site decreased in all fractions compared to corresponding fractions of the C25 site. There was no significant difference in the δ^13^C values in the densest fractions of the forest soil F5, F12 compared to corresponding fractions of C5, C12, respectively.

### SOC derived from forest litter C of density fractions

**Fig 9:**
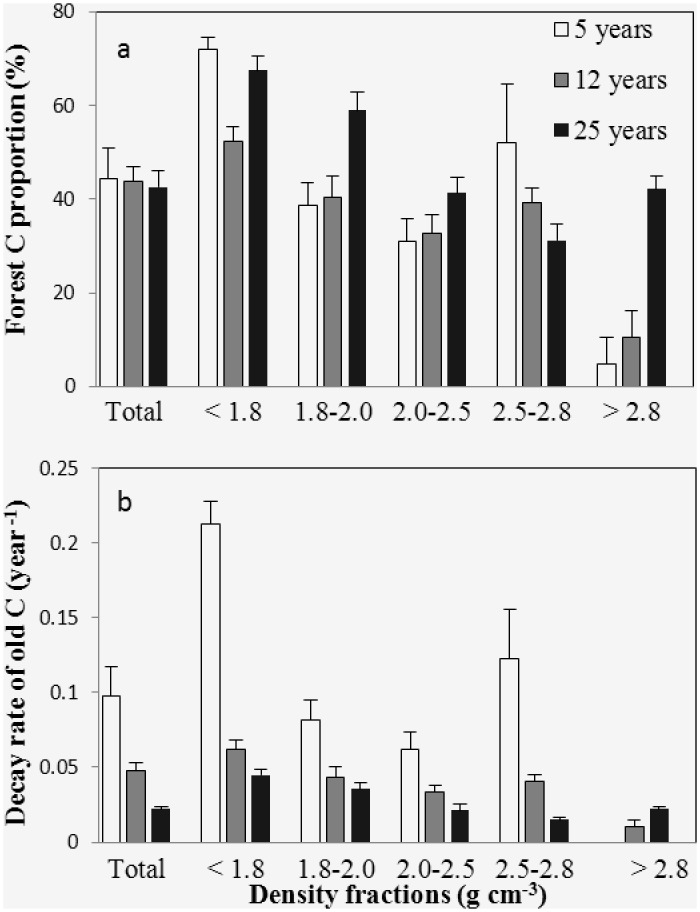
Forest C input (a) and decay rate (b) of old C (mean ± standard error) for bulk soil and density fractions of 5, 12 and 25 years forest.

The stable isotope analysis indicated that δ^13^C abundance in the forest soil was more depleted than that in the crop soil (Table 2). The greastest forest C contribution was found in the < 1.8 g cm^-3^ fractions (Fig. 9a). The fastest decay rates of crop C were found in the < 1.8 g cm^-3^ fractions of 5 years forest (Fig. 9b). The forest C proportion showed decreased trend from the lightest fraction to 2.0–2.5 g cm^-3^ fractions. The decay rates of crop C showed similar trend from the lightest fraction to 2.0–2.5 g cm^-3^ fractions.

## Discussion

Afforestation resulted in a significant decrease in SOC concentration at the 5 years forest sites and increase in SOC concentration in 12 and 25 years sites, compared to crop sites, respectively. Our results, agreed with previous studies that there was an initial decrease in SOC after afforestation followed by a gradual increase in SOC [10–12]. SOC sequestration following afforestation is determined by the balance between forest litter input and soil OC decomposition [7, 32]. The shifts in microclimate, lack of additional fertilizer provided for young plants, and the changes in quantity and quality of C input changes generally contributed to accelerated mineralization of old C in younger forest [7, 8, 33, 34]. At 5 year old forest, high decay rates, as confirmed by the highest old C decay rates, offset the litter input rates, which led to decrease in SOC concentration. There are reports that after establishment of forest, it requires at least 5 years the litter inputs starts to accumulate [9, 35]. The increase of SOC content in 12 and 25 year old forests were likely induced by the decreased decay rates and the increased litter inputs, as confirmed by the increased C/N and decreased δ^13^C in the 25 year old forest.

The high peak values for the mass distribution of 2.5–2.8 g cm^-3^ fractions were due to the soil sandy texture and mineralogy composition. The minerals included quartz (2.65 g cm^-3^), albite (2.6–2.7 g cm^-3^) and microcline (2.53–2.56 g cm^-3^). Afforestation of the system did not affect the density fraction weight distribution when compared to the adjacent crop soil. This is in agreement with Six et al as the aggregate distribution did not change between forest and crop soil [36].

Density fractions showed different mineralogy composition, OC composition, and dynamic characteristics in this study. High C/N in the < 1.8 g cm^-3^ fractions indicated that the coarse plant debris in this fraction had been partly decompsed because the C/N of this fraction was lower than fresh litters [21, 37]. This fraction from 12 and 25 yr forest showed higher C/N and lower δ^13^C compared to other fractions, and held the highest forest C propotion and the highest decay rates of old C. These results indicated that forest litter derived C mainly entered into these fractions and the SOC turnover rates was higher in these factions than others. Other studies reported similar results where the OC in the light fraction had relatively higher turnover rates than the heavy fractions [38, 39].

There are studies about radiocarbon dating suggest that C associated with denser particles typically has a greater mean residence time [22]. We observed crop C associated with 1.8–2.5 g cm^-3^ fractions was depleted at a slower rate than C associated with lightest fractions. This indicated that SOC sorbed or entrapped within microaggregates and sorbed directly on mineral surfaces are not bioavailable for microorganisms, due to decreased accessbility, and strong association between organic C and the mineral [40–42]. Higher contribution to total C content of 2.0–2.5 g cm^-3^ fractions than other fractions which indicates that microaggregate protection and association between organic material and minerals provided a major source of contribution for the OC sequestration within the afforested soil system. The decline of C/N in forest soil fractions from 1.8–2.0 g cm^-3^ to 2.0–2.5 g cm^-3^ indicated that C was progressively decomposed due to microbial metabolic processes [21, 22, 43, 44]. Studies have shown that aggregates formed due to plant-mineral association is susceptible to breakdown and recombination by mesofauna and microorganism activity [37, 45]. The decreasing trend of the forest C proportion from the 1.8–2.0 g cm^-3^ fractions to the 2.0–2.5 g cm^-3^ fractions is possibly due to the fact that incorporation of forest litter-derived C occurred from coarse plant debris of low density (< 1.8 g cm^-3^) to aggregates of higher density (1.8–2.5 g cm^-3^) through aggregate recombination [15, 40, 46] and soluble OC transport through the pore system of aggregates [47].

Our XRD analyses revealed that the 2.5–2.8 g cm^-3^ fractions consisted of crystallized primary minerals, mainly quartz, albite and microcline, but fractions > 2.8 g cm^-3^ were comprised of quartz, albite and clinochlore and amphibole. Clinochlore and amphibole are minerals that offer more potential reactive sites for SOM adsorption than primary minerals such as quartz and albite [40]. Studies have shown that mineral-associated SOM exhibit C/N ratios of 7–14 [48]. The densest fraction (> 2.8 g cm^-3^) in this study exhibited C/N of 2–4, indicating N enrichment of SOM adsorbed in this fraction. Highly nitrogenous compounds, such as proteins, could be preferentially adsorbed by reactive mineral surfaces [49]. At the three forest sites, forest C contributions were observed in the 2.5–2.8 g cm^-3^ and > 2.8 g cm^-3^ fractions, which probably came from direct adsorption or OC transfer from the light fractions. Some studies have shown that decomposed C and microbial byproducts with amphiphilic characteristics could adsorb or diffuse directly on to the mineral associated fraction or transfer from the light fractions to the mineral surface through pore system within the aggregates [37, 45, 50].

## Conclusions

Afforestation resulted in significant decrease in SOC concentration for the 5 year old forest and increase in SOC concentration in the 12 and 25 year old forest. Afforestation had a significant impact on C dynamics in the density fractions. However, it did not affect the soil mass, SOC, and N proportional weight distributions when compared to the adjacent crop soil. Incorporation of forest litter-derived C occurred in a range of densities, from the low density (< 1.8 g cm^-3^) fractions to aggregates of higher density (1.8–2.5 g cm^-3^) through aggregate recombination and C transport in the pore system of the aggregates. During this process, fresh C progressively decomposed due to microbial processes. Some forest litter-derived C could transfer from the light fractions or directly diffuse and adsorb onto mineral particles. Microaggregate protection and association between organic material and minerals provided major contribution on the SOC sequestration within the afforested soil system.
